# *In Vitro-In Vivo* Correlation Evaluation of Generic Alfuzosin Modified Release Tablets

**DOI:** 10.5402/2012/813836

**Published:** 2012-11-20

**Authors:** Utpal Kumar Sanki, Badal Kumar Mandal

**Affiliations:** Environmental and Analytical Chemistry Division, School of Advanced Sciences, VIT University, Vellore 632014, India

## Abstract

Alfuzosin, a selective alpha-1a antagonistis is the most recently approved AARAS, with limited cardiac toxicity and exclusively used for lower urinary tract syndromes (LUTS). In order to reduce pill burden and better patient compliance modified release (MR) formulations have been developed. Alfuzosin MR tablet was developed by the use of hot-melt granulation techniques using mono- and diglycerides as rate controlling membranes to minimize health care cost and uses of costly excipients. The other purpose of the study was to evaluate *in vitro-in vivo* performance of the scale up batch in healthy human subjects for commercialization. The blend uniformity (mean ± RSD%), assay, cumulative percent dissolution at 24 h, hardness, and friability of the biobatch were 100.2 ± 0.05%, 100.43 ± 0.023%, 93.98%, 4.5 kg, 5 min, and 0.08%, respectively. The *in vivo* pharmacokinetic parameters under fasting conditions between test and reference formulations (Uroxatral 10 mg extended release tablets) were comparable. The 90% CI, geometric mean ratio (%) and power of *C*_max_, AUCT, and AUCI of the fasting study for the test and reference formulation were 99.03% to 122.78%, 109%, 0.998; 92.94% to 116.71%, 104%, 1; 98.17% to 124.01%, 110% 1, respectively. The scale up biobatch showed negligible difference in *in vitro* properties with respect to the pilot batch. The formulation developed with these agents was safe to use as there were no serious adverse events developed during the conduction of the clinical trial on the healthy subjects. Furthermore, the developed formulation was bioequivalent with respect to rate and extends of absorption to the reference formulation.

## 1. Introduction 

The chronic prostatitis syndromes and symptomatic benign prostatic hyperplasia (BPH) are common among aged men and women [1]. The etiology of prostatitis syndrome remain elusive, it does appear to be associated with a high incidence of voiding dysfunction [2]. Lower urinary tract syndromes (LUTS) are sometimes associated with enlarged prostate, commonly referred to as BPH. BPH can exist without LUTS. The BPH sometimes causes blocks of bladder outflow [3] which cause pain and inflammation of the urinary tract. If untreated they can cause impair urinary frequency, nocturia, incomplete emptying, and urinary hesitancy.

In the past, the treatment of LUTS, associated with clinical BPH, was restricted to surgical interventions, such as transurethral resection of the prostate or open nucleation of the enlarged adenoma [4]. In the last decade, however, minimally invasive treatment as well as noninvasive treatment options have been explored and developed, many of them based on the administration of heat to the enlarging adenoma and administration of the drug therapy including alpha-blockers and 5 alpha-reductage inhibitors. Selective alpha-adrenergic receptor antagonists (AARAs) such as prazosin, terazosin, doxazosin, and tamsulosin are important in the treatment of symptomatic Benign prostatic hyperplasia (BPH) [4, 5]. Among these AARAS only tamsulosin is uroselective which selectively blocked alpha-1a receptor (alpha-1a predominate in the urinary tract and 1b in the vasculature). Therefore all AARAS are suspected for the cardiovascular side effects as they all blocked the alpha-1b.

Alfuzosin is the most recently approved AARAS, with limited cardiac toxicity in the United States for symptomatic treatment of BPH. Alfuzosin, selective alpha-1a blockers [6] differs from other AARAS by the absence of a piperidine moiety and the presence of a diaminopropyl spacer, which confers alfuzosin with specific biochemical properties [7].

 Alfuzosin is currently marketed throughout Europe, Asia, and Latin America exclusively for the treatment of symptomatic BPH. There are 2 bioequivalent formulations available [8]: an immediate release form (2.5 mg, 3 times daily) and a sustained-release form (5 mg, 2 times daily and 10 mg once daily). The efficacy of both formulations has been demonstrated in well-designed placebo-controlled studies [9, 10]. The onset of action of alfuzosin is rapid from the first dose and it maintains symptom relief for up to 3 years [11]. Alfuzosin is highly water soluble [12] and after fasted conditions its oral bioavailability was increased by 40% in consumption of 25% food more than the normal food intake and *C*_max⁡_ by 50% [13].

A once-daily formulation, which delivers alfuzosin *via* a novel prolonged-release system, has been developed to improve the convenience of dosing and to provide optimal pharmacokinetic coverage over 24 h [14, 15]. Since the drug is highly water soluble (BCS class 1 drug) [16], controlling its release from the dosage forms is the major challenge to fabricate controlled release formulation [17]. In order to control its release from the dosage forms, present marketed reference listed drug (RLD) exploited many pharmaceutical rate controlling polymers, namely, hydroxy propyl ethyl cellulose, microcrystalline cellulose, ethylcellulose, and hydroxy propyl methyl cellulose to control release of highly soluble alfuzosin [11]. 

Therefore the aim of the present formulation development was to prepare control release alfuzosin 10 mg tablets by hot-melt extrusion process with the use of mono- and diglycerol as a rate controlling membrane. The other aim of the study was to match the *in vitro* characteristics and* in vivo* performance of the formulation using bioequivalence study in the healthy volunteers to establish bioequivalence with respect to RLD Uroxatral.

## 2. Methods and Materials

### 2.1. Materials

Alfuzosin hydrochloride was obtained from the Dr. Reddys Laboratory, Hyderabad, India. Marketed product was obtained from the local pharmacy shop. Mono- and diglycerides NF (lmwitor 900) were donated from Hetero Lab, Hyderabad, India. Lactose monohydrate NF was procured from Medreich labs, Bangalore, India. The purified talc USP (Luzenac Corp.) and magnesium stearate NF were purchased from Signet Chemical Corporation Pvt. Ltd, Mumbai, India and colloidal silica (aerosol 200) was purchased from Evonik Degussa India Pvt. Ltd. Mumbai, India. Opacode Black 8-1-8152 HV was collected from Colorcon India limited. Working standards were procured from Zydus Cadila Healthcare Ltd. Gujarat, India. K3EDTA containers, disposable syringe, and ria vial were supplied by BD, India. 

### 2.2. Methods

**Excipients Compatibility Screening**

The excipients were selected based on the results of compatibility study which were further confirmed by comparing the excipients used by reference listed drug. The compatibility study was conducted at 40°C/75% RH for 4 weeks. The closed glass vial containing physical mixture of alfuzosin hydrochloride and excipients in a particular ratio were subjected at 40°C/75% RH for 4 weeks to determine the change of related substances as compared to the original API (Active pharmaceutical agents). If total impurity of API was increased to more than 1%, then it was concluded to have interaction of those excipients with the drug.

**Selection of Excipients and Their Grades**

The grade of mono- and diglycerides used was of NF grade (Imwitor 900, Sasol). The quantity of mono- and diglycerides was used in concentrations up to 45% as a rate controlling agent. Trials were carried out using the excipients in concentrations of 10.0%, 20.0%, 30.0%, 40.0%, 50.0%, and 60.0% in hot melt granulation process to get the desired release profile with some quantity being added intragranularly and the other quantity added extragranularly. The grade of colloidal silicon dioxide used was of pharmaceutical grade (Aerosil 200) with concentrations up to 3.0% as a lubricant. The grade of talc and magnesium stearate used was of pharmaceutical grade and their quantities were chosen based on requirements.

**Formulation Developments**

In order to establish the robustness of the proposed formulation, and to optimize the rate controlling polymer concentration, the following ranges around the target formulation were investigated in the experiments. The level of various rate controlling membranes at different proportion is given in Table 1.

Alfuzosin hydrochloride and lactose monohydrate were passed through no. 40 mesh; mono- and diglycerides was sifted through no. 20 mesh and blended together in jacketed Rapid Mixer Granulator (RMG) for 10.0 min with intermittent raking of the mixture. The hot-melt granulation of the blend was carried out by passing the steam or hot water through jacket of the RMG until the temperature of the blend reached to 70°C and the entire dry blend converted into molten mass [18, 19]. The molten mass was cooled at room temperature by passing cold water through jacket and the dried granules were sifted with colloidal silicon dioxide through 1.2 mm screen. Blended granules were lubricated by no. 40 meshes passed talc for 15.0 min and then lubricated with magnesium separate no. 40 mesh (presifted) for 5.0 min. The lubricated blend was taken for compression by 10/32′′ Beveled edge SC punches, plain on both sides to the target weight of 225 mg. The details of manufacturing process and in process quality control used for the product development are given in Figure S1 (see Supplementary Material available online at doi:10.5402/2012/813836).

Six different concentrations of rate controlling agents were formulated in six different tablets formulations of same hardness and dissolution of all formulations were carried out using USP dissolution apparatus type II under nonsink conditions in 900 mL HCl (0.1 N). Similarly, different hardness of 6 formulations were prepared and the dissolution of the drugs were tested using USP type II dissolution apparatus in 900 mL HCl (0.1 N).

### 2.3. *In Vitro* Assay

20 tablets were grinded using a mortar and pestle and the mixture equivalent to 10 mg drug was weighted. Mixture was then transferred to 100 mL volumetric flask and dissolved with small quantity of water followed by 10 min settling. 10 mL of the solution was diluted further to 100 mL in a 100 mL volumetric flask.

**Scale up of Alfuzosin Hydrochloride MR Tablets**

Scale up in the jacketed RMG and 27 station compression machines were successfully accomplished for the drug product placebo. Additionally based upon the design of experiments on laboratory scale batches, acceptable ranges for critical process parameters for melting, mixing, and compression were determined. This process knowledge was used to successfully scale up from the laboratory scale to pilot scale in the production of the pivotal batch [20, 21]. The blend for the same was carried out in 25 liter jacketed RMG involving granulation, cooling, sifting and milling followed by blending and lubrication in 1 lot. The lubricated blend was compressed to 13300 tablets.

The molten masses was then chopped with fast chopper speed and slow mixing for 2.0 min. Hot granules were cooled down for the 2 h at room temperature. Milling of the resultant granules was done through oscillating granulator with a screen size of 0.8 mm nearly 9 kg blends. Finally extragranular ingredients were blended through a conta blender (30/60/120 L capacity) for 20 min. with a speed of the motor is 15 rpm [22, 23]. The blends were taken out at 10, 15, and 20 min for analysis of blend to establish blend uniformity. All the above parameters were kept constant of the pilot and pivotal batches of the formulations except screen size was increased to 1.2 mm instead of 0.8 mm [24] (Table S1 in Supplementary Material).

**In Process Testing Procedure**

1 g of blend in a clean dry Petridis was taken and observed visually against black background. UV absorption spectrum of sample preparation exhibited maxima and minima, which were at the same wavelengths as that of standard preparation obtained in the assay. Standard preparation and sample preparation were stable at room temperature up to 24 h. Blend analysis was conducted by sampling the mixture in the intermediate bulk containers (IBCs) that is, in stainless steel bunkers (containers).

**Sample Preparation**

The alfuzosin hydrochloride was weighed accurately in a 100 mL volumetric flask containing 10 mL alcohol and was sonicated for 30 min with occasional shaking. The volume was made up to the mark with methanol diluents and diluted to a known concentration of about 0.005 mg per mL of alfuzosin hydrochloride. The solution was filtered through 0.45 *μ*m Millipore PVDF filter. The same procedure was adopted for all the samples of different locations.

**Procedure**

The absorbance of standard preparation and sample preparation was measured at 244 nm against diluents as blank (methanol) [25, 26]. The method was validated if the absolute difference of the standard reading at the initial and end of the run was not more than 2.0%. The quantity of alfuzosin hydrochloride (in %) for each location was calculated using the following formula 1 as (ATi/AS)×(WS/100)×(2/100)×(DTi/W3i)×(*P*/100)×(Ave.  wt./10) × 100%, where ATi = absorbance of sample preparation (*i* = 1 to 10); AS = absorbance of standard preparation; WS = weight of working standard taken in mg; W3i = weight of sample taken in mg (*i* = 1 to 10); DTi = dilution of sample preparation (*i* = 1 to 10); *P* = percentage purity of working standard. The results of 10 locations were taken for calculation of mean and RSD.

### 2.4. Assay of Blend

**Standard Preparation**

25 mg of Alfuzosin hydrochloride working standard was transferred to a 100 mL volumetric flask and dissolved in methanol by sonication. Then 2.0 mL of this solution was diluted to 100.0 mL with diluents (methanol) for analysis by spectrophotometer with a *λ*_max⁡_ of 244 nm. The blend equivalent to 30 mg of alfuzosin hydrochloride was dissolved in 500 mL volumetric flask using methanol by sonication with occasional shaking for about 30 min, filtered through 0.45 *μ*m Millipore PVDF filters and 4.0 mL of this filtrate was diluted to 50.0 mL with methanol for analysis. The quantity of alfuzosin hydrochloride (in %) per blend for both sample preparations was measured using the formula 2 as (ATi/AS)×(WS/100)×(2/100)×(500/WTi)×(50/4)×(*P*/100)×(Ave.  wt./10) × 100, where, ATi = absorbance of sample preparation (*i* = 1 and 2); AS = absorbance of standard preparation; WS = Weight of working standard taken in mg; WTi = Weight of sample taken in mg (*i* = 1 and 2); *P* = percentage purity of working standard.

**Pivotal Batch of Alfuzosin Hydrochloride Tablets**

The pivotal batch of alfuzosin hydrochloride ER tablets 10 mg was planned to make 130,000 tablets. The hot-melt granulation was carried out in 25 L jacketed Rapid Mixer Granulator involving granulation in 3 lots, cooling in 3 lots, sifting, milling followed by blending and lubrication in 1 lot [27, 28]. The lubricated blend, after release was compressed to 130,000 tablets. The target hardness, thickness, friability, diameter, and machine speed were kept 4.5 kb, 4.5 ± 0.5 mm, NMT 1.0%w/w, 7.9 ± 0.2 mm, and 20 ± 10 rpm, respectively [28]. These parameters were kept constant for all commercial batch productions. The compositions of various batches preparation is given in Table 1.

### 2.5. *In Vitro* Assay and Content Uniformity Test

**Preparation of Standard Drug Samples**

20 mg of alfuzosin hydrochloride working standard was dissolved in 75 mL of mobile phase (mixture of buffer (pH 3.5): acetonitrile at a ratio of 4 : 1) using sonication. 10.0 mL of this solution was mixed with 50.0 mL mobile phase (mixture of buffer (pH 3.5): acetonitrile at a ratio of 4 : 1).

**Preparation of Assay Samples**

20 tablets were weighed and grinded in a mortar and pestle. The mixture equivalent to 20 mg of the drug was transferred into 100 mL volumetric flask containing 75 mL of mobile phase and sonicated to dissolve. Then 10.0 mL of this solution was diluted to 50.0 mL with diluents and mixed. The buffer solution was prepared after mixing 5 mL of 70% perchloric acid with 1000 mL deionized water and finally pH was adjusted to 3.5 ± 0.05 with 10(N) sodium hydroxide.

**Preparation of Content Uniformity Samples**

Each tablet was transferred to 100 mL volumetric flask and dissolved in mobile phase after 20 min sonication. Then 20 mL of the above solution was diluted to 50 mL with mobile phase.

**Procedure**

Separately 20 *μ*L mobile phase, standard preparation, and sample preparation were injected into Waters HPLC system equipped with Kromasil Cl8 (Ll) (150 mm × 4.6 mm, 5 micron) column at 25°C and run for 20 minutes at a flow rate of 1.5 mL/min and *λ*_max⁡_ of 254 nm using UV detector to collect response. The percentage assay (assay%) on anhydrous basis for both the sample preparations was calculated using the following formula 3 as (ATi/AS)×(WS/100)×(10/50)×(100/WTi)×(*P*/100)×(100/[100 − %water]) × 100, where ATi = peak area of sample injection (i = 1 and 2); AS = peak area of standard injection; WS = weight of working standard taken in mg; WTi = weight of sample taken in mg (*i* = 1 and 2); *P* = percentage purity of working standard.

### 2.6. Determination of API Impurities in the Finished Dosage Forms

**Resolution Solution**

20 mg of alfuzosin hydrochloride working standard and 20 mg of impurity A standard was mixed with 75 mL diluents and made to 100 mL after sonication. 1.0 mL of this solution was diluted to 100.0 mL and injected to HPLC. Impurity solutions were prepared by dissolving 15 mg of each impurity in 100 mL diluents and 10 mL of it was diluted to 50 mL for analysis. Composite impurity solution was prepared by mixing 1 mL of each impurity solution to 100 mL standard solution containing 20 mg Alfuzosin hydrochloride (AFH). Sensitivity solution was prepared by 800-fold dilution of AFH. System suitability was evaluated using sensitivity solution as follows: signal to noise ratio (SNR) be more than 10; resolution between AFH and impurity A be more than 3; column efficiency for analyte peak be more than 2000 theoretical plates; the tailing factor for analyte peak be less than 2 and relative standard deviation for 5 replicate standards be less than 1.0%. The peak of the corresponding impurities was identified [29]. The area of the peak for each impurity was calculated and finally estimated the % of known impurity present in the formulation by the formula 4. (AT/AS)×(WS/100)×(10/50)×(100/WT)×(*P*/100) × 100 × RRF, where AT = peak area of corresponding unknown impurity in sample injection; AS = peak area of Alfuzosin hydrochloride in standard injection; WS = weight of working standard taken in mg; WT = weight of sample taken in mg; *P* = percentage purity of working standard and % of total impurities = sum of % all impurities (known + unknown).

**Determination of Residual Solvents in Dosage Forms**

Dimethyl sulfoxide as a diluents was used to determine residual solvents like methanol, ethanol, diethyl ether, acetone, isopropyl alcohol, dichloromethane, ethyl acetate, tetrahydrofuran in the finished dosage forms using method described elsewhere [28]. Stock solution A was prepared by mixing 30 mg methanol, 50 mg ethanol, 50 mg diethyl ether, 50 mg acetone, 50 mg isopropyl alcohol, and 50 mg ethyl acetate standard into a 100 mL volumetric flask containing 25 mL of diluents. Stock solution B was prepared by mixing 60 mg dichloromethane and 72 mg tetrahydrofuran standard into a 100 mL volumetric flask containing about 25 mL of diluents. Stock solutions A and B were mixed in a ratio of 5 : 1 (*v/v*) in diluents for combined standard. In brief, the working procedure is as follows. 50 mg of sample was taken in a 20 mL head space vial with 5.0 mL of diluents. Supelco Capillary column (30 m × 0.53 mm, 3 *μ*m) was used at nitrogen carrier gas flow rate of 2.0 psi. The initial column temperature was kept for 35°C and increased 25°C/minute thereafter to reach the final column temperature of 225°C while the injection port temperature and detector temperature were kept 230°C and 250°C, respectively. System suitability was considered to have relative standard deviation for six replicate standard injections not more than 15.0% against each solvent. The residual solvent content in the dosage forms (in ppm) was calculated using the equation 5 [27] as (AT/AS)×(WS/100)×(10/100)×(5/WT)×(*P*/100) × 10^6^, where AT = peak area of corresponding solvent in sample injection; AS = peak area of corresponding solvent in standard injection; WS = weight of corresponding solvent standard taken in mg; WT = weight of sample taken in mg; *P* = percentage purity of corresponding solvent standard.

### 2.7. *In Vitro* Dissolution Study

In order to establish release patterns of the drug from the tablets formulation, a dissolution study was conducted for a period of 22 h using USP dissolution apparatus II (paddle) under nonsink condition [30, 31] equipped with autosamplers. The dissolution media was 500 mL of 0.1 N HCl, for comparing the release rate among the trial lab scaled batches and to establish the reproducibility of the release rate from the formulation. The formulation which showed reproducible dissolution behavior was considered as final formulation and taken for the further scale up study. In addition 900 mL of dissolution media each of 0.1 N HCl (pH 1.2), acetate buffer (pH 4.5), and phosphate buffer (pH 6.8) were used to compare final scale up formulation with innovator formulation. During dissolution, the dissolution media were maintained at 37 ± 0.5°C and paddle speed was 100 rpm at pH 1.2 and 4.5 whereas 50 rpm at pH 6.8. Samples through a 40 *μ*m filter were injected automatically at each sampling time point [32]. Alfuzosin release was detected by Shimadzu UV spectrophotometers absorbance at 244 nm using. 22 mg of alfuzosin hydrochloride working standard was dissolved in 5 mL methanol and made the volume up to 200 mL with dissolution medium. 20-fold diluted sample was injected to instrument for analysis. The quantity of alfuzosin hydrochloride (in %) released was quantified by using formula *x* as (AT/AS)×(WS/200)×(900/10)×(10/5)×(P/100) × 100 at 1 h. Net/cumulative drug dissolution was calculated at each time point after adding drug present in the volume of sample taken out as correction factor.

### 2.8. Bioequivalence Study

Forty six healthy male Indian volunteers aged between 18 and 45 years with BMI (body mass index) within 19 to 29.4 kg/m^2^ enrolled for the study after health clearance by general physical examination and clinical lab evaluation (including ECG and chest X-ray). The normal value ranges of the following laboratory tests: albumin, alkaline phosphatase, AST, ALT, blood glucose, creatinine, *μ*-GT, total bilirubin, total protein, triglyceride, total cholesterol, haemoglobin, hematocrit, total and differential white cell counts, routine urinalysis, and negative serology for HIV, HBV, and HCV were determined before enrolling them in the present study. All the subjects gave written informed consent and Madras Ethics Committee, Chennai, Tamil Nadu, India approved the clinical study protocol. The study was conducted in accordance with ICH GCP, Indian GCP, and ICMR guideline with the provisions of the Declaration of Helsinki (Seoul 2008).

The study was an open labeled, randomized, two sequence, two periods, single-dose, two-way crossover design with 14 days washout period between the doses [13, 33]. During each period, the volunteers were housed in a clinical pharmacology unit of Huclin Research Limited, Chennai, Tamil Nadu, India on the eve of the dosing day. Following over night fasting of about 10 h a single dose of alfuzosin (10 mg CR tablets of either test or reference formulation) was administered orally to each of the volunteers as per randomization code list at sitting posture with the aid of 240 mL of water in a staggered manner in order to easy sample collection. A mouth check following drug administration with the help of a tongue depressor was performed for each of the volunteers to ensure the compliance of the dosing activities. All the subjects were restricted to 2 h after drug administration of water intake and toileting, while at other time water was given ad libitum. A standard meal was provided to all the volunteers at 4, 8, and 12 h after drug administration. Vitals were recorded at 2, 4, 6, 8, 12, and 24 h after drug administration for each volunteer to assess health related complication if any. No other food was permitted during the “in-house.” Serial blood samples were collected from each of the volunteers at every period at predose and at 1.00, 2.00, 3.00, 4.00, 5.00, 5.500, 6.00, 8.00, 10.00, 12.00, 16.00, 24.00, 36.00, 48.00, and 72.00 h post drug administration. Collected blood samples were centrifuged at 3500 rpm for 10 minutes at temperature 10°C to collect plasma. Plasma samples were kept in RIA vial for bioanalysis. A validated LC-MS/MS method was used to estimate the concentration at each time point for all the subjects. The subjects' data were subjected to noncompartmental analysis to determine the pharmacokinetic properties using WinNonlin v5.3. Log transformed pharmacokinetic data were subjected to multivariate ANOVA analysis to construct point estimate (geometric mean ratio between test and reference) and 90% CI using two one-sided test to establish bioequivalence. As per bioequivalence guideline of generic product, 90% CI should have a predefined range of 80 to 125% with respect to reference in order to establish bioequivalence with a nominal power of at least 80%.

### 2.9. Determination of Alfuzosin in Human Plasma

A method for determining alfuzosin in human plasma was validated using an API 3000 LC/MSIMS system with detection in the range of 0.05 to 30.00 ng/mL and Aquacil C18 column (100 × 2.1 mm, 5 *μ*m) was attached for separation [34, 35]. The interface used with the API 3000 LC MS/MS was a TurbolonSpray. The positive ions were measured in MRM mode. The analytes were quantitated using a solid phase extraction method. Each 500 *μ*L aliquot of standard and QC samples were mixed with 25.0 *μ*L of deionized water. 0.2 mL of internal standard (IS) working solution (20.0 *μ*g/mL) was added to the entire sample collected from volunteers except blank samples. The sample was applied to prewashed SPE cartridges (Waters Oasis, 1 mL) with 1.0 mL of methanol followed by 1.0 mL of deionized water and centrifuged for l min at 3000 rpm at each step. Each cartridge was washed with 1.0 mL of deionized water by centrifugation for 2 min at 3000 rpm. The sample was eluted from Oasis cartridge by adding 1.0 mL of methanol and centrifugation for 2 min at 3000 rpm. The eluent was evaporated to dryness at 40°C under gentle stream of nitrogen. The residue was reconstituted in 300 *μ*L of reconstitution solution and 5.0 *μ*L was injected onto a LC-MS/MS system.

### 2.10. Pharmacokinetics and Statistical Analysis

The pharmacokinetic parameters were determined from the plasma concentration and time data using a noncompartmental analysis by WinNonlin professional software (Version: 5.3; Pharsight Corporation, USA). The log transformed pharmacokinetic data were subjected to ANOVA and two one side *t*-test analysis to estimate geometric mean ratio, 90% CI and power by the use of SAS V 9.3. As per bioequivalence guideline, [33, 36] 90% CI will be constructed, which will be predefined values of 80 to 125% of the reference drug in log scale to establish generic equivalence.

### 2.11. *In Vivo-In Vitro* Correlation (IVIVC) Study

IVIV link model was used to build correlation; *in vitro* fraction of drug released at each time points was subjected to Weibul model fit options in IVIVC tool kit of WinNonlin v 5.3. The individual subject *in vivo* data for both test and reference were fitted to Wagner Nelson model to calculate the relative fraction of the drug absorbed *in vivo*. Unit impulse response of *in vivo* data followed by deconvolution of the data was used to build correlation with *in vitro* dissolution data [36]. The correlation coefficient, prediction error of *C*_max⁡_ and AUCT were calculated across all the dissolution media to conclude the discriminative dissolution media for the drug.

## 3. Results

### 3.1. Formulation

Drug excipients compatibility study was carried out to find out status of impurities present in the formulation (Table S2 in Supplementary Material). There was no increase in impurities, no change in the physical appearance of the formulation. Hence it was concluded that mono- and diglycerides, lactose monohydrate, colloidal silicon dioxide, magnesium stearate, and talc were compatible with alfuzosin hydrochloride in the formulation. 

The generic alfuzosin 10 mg ER tablets were prepared by hot-melt granulation using mono- and diglycerides at various proportions used as a rate controlling membrane (Table S3 in Supplementary Material). The list of other ingredients used in the development of the formulation is given in Table 1. Various pilot batches were trialed to optimize drug release, hardness and friability (Table 2), and the optimized formula (F025) was chosen based on *in vitro* dissolution study which corresponds to the reference dissolution among pilot formulations.

The blend uniformity was studied as part of in-process test during the manufacturing of 10 mg alfuzosin hydrochloride extended release tablets. Blend analysis was conducted by sampling the mixture in the intermediate bulk containers [34] (IBCs) that is, stainless steel bunkers (containers). Results showed uniform distribution of the drug among top, middle and lower portion of the blend with a insignificant departure of 0.23% (Tables S1 and S4 in Supplementary Material).

The assay of 6 individual tablets passed the USP limit of 90 to 110% of drug (Table S4 in Supplementary Material) with a relative standard deviation of 0.023. The hardness of the pivotal and commercial batch was kept for 4.5 kp (Kilo Pascal). The observed hardness (mean ± SD kp, *n* = 20) of the pivotal batch was 4.52 ± 0.23 and that of commercial batch was 4.53 ± 0.31 (Table S4 in Supplementary Material). The friability of the all batches was found to be less than 1% (0.8% for biobatch and 0.84% for commercial production batch) (Table S4 in Supplementary Material).

**Figure 1:**
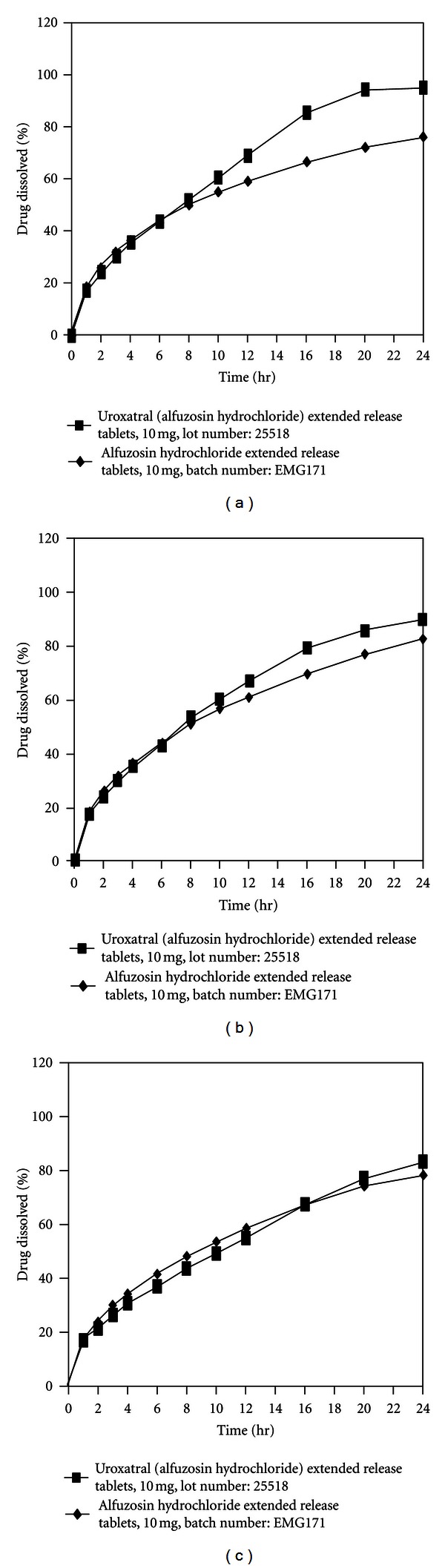
*In vitro* comparative dissolution profile of reference drug Uroxatral (alfuzosin hydrochloride) extended release tablets 10 mg versus test drug alfuzosin hydrochloride extended release tablets 10 mg. Dissolution media is 0.1 (N) hydrochloric acid (a), acetate buffer (b), and phosphate buffer (c). Dissolution volume: 900 mL; apparatus USP-II (Paddle); RMP = 100; temperature (37°C ± 0.5°C).

The drug release from the biobatch was identical for the first few hours and got difference in the last hours of the cumulative drug release with respect to reference formulation. The cumulative percentage drug release in 0.01 N HCl (pH 1.2) (Figure 1(a)), acetate buffer (pH 4.7) (Figure 1(b)) and phosphate buffer (pH 6.8) (Figure 1(c)) in 900 mL media for both biobatch and reference formulation. The cumulative percent drug release of the biobatch decreased to 95% against 96.99% at 24 h in 0.1 N HCl (pH 1.2) (Tables S5–S10 in Supplementary Material) [36]. 

### 3.2. Bioequivalence Study

35 out of 42 subjects completed both the periods of the fasting study and there were 7 discontinued subjects. 2 subjects were prematurely withdrew their consent without any reason, 3 subject discontinued due to severe headache and other two subjects did not report for the second period to the facility. However such complications were resolved without squeal. There were no clinically significant changes in vital signs, clinical laboratory variables, ECG, X-ray and general physical examination for fasting study.

### 3.3. Determination of Alfuzosin in Human Plasma

The data was acquired by and calculated on Applied Biosystems “Analyst” version 1.4.1 Software. Linear regression, with l/x^2^ weighting, was used to obtain the best fit of the data for the calibration curves (figure not shown). The lower limit of quantitation (LLOQ) was 0.05 ng/mL and the upper limit of quantitation (ULOQ) was 30.00 ng/mL. Quality control samples (six sets) at concentrations of low 0.1500 ng/mL (LQC), medium 2.000 ng/mL (MQC), and high 25.00 ng/mL (HQC) prepared in human plasma, were analyzed with each assay validation run to ensure acceptable assay precision and accuracy. Also six sets of LLOQ (0.050 ng/mL) and ULOQ (30.00 ng/mL) samples included in each batch were run. In addition, the stability of alfuzosin during freeze thaws cycles, extracted samples in the refrigerator and on bench top, in biological matrix, stock solution stability at room temperature and 4°C ± 6°C and intermediate stock solution stability for alfuzosin at 4°C ± 6°C was studied. The overall interday precision (%CV) and accuracy for the standards and quality control samples were 1.4 to 6.0% and 96.1 to 104%, respectively. The interday precision and accuracy for the LLOQ for alfuzosin were 9.7% and 102%, respectively, and for the ULOQ were 5.1% and 103%, respectively (Table 3).

Method did not show any matrix effects since the accuracy of the method was 103% at highest calibration concentration. The recovery of the internal standard was about 68% (Table 3). The highest accuracy was also obtained from the diluted standard which was 96.9% (Table 3), bench top stability and stock solution stability (Table S11 in Supplementary Material) and freeze thaw stability (Table S11 in Supplementary Material) and room temperature stability (Table S11 in Supplementary Material) were also in acceptable limit which signified the validity of the method.

### 3.4. Pharmacokinetics and Statistical Analysis

**Figure 2:**
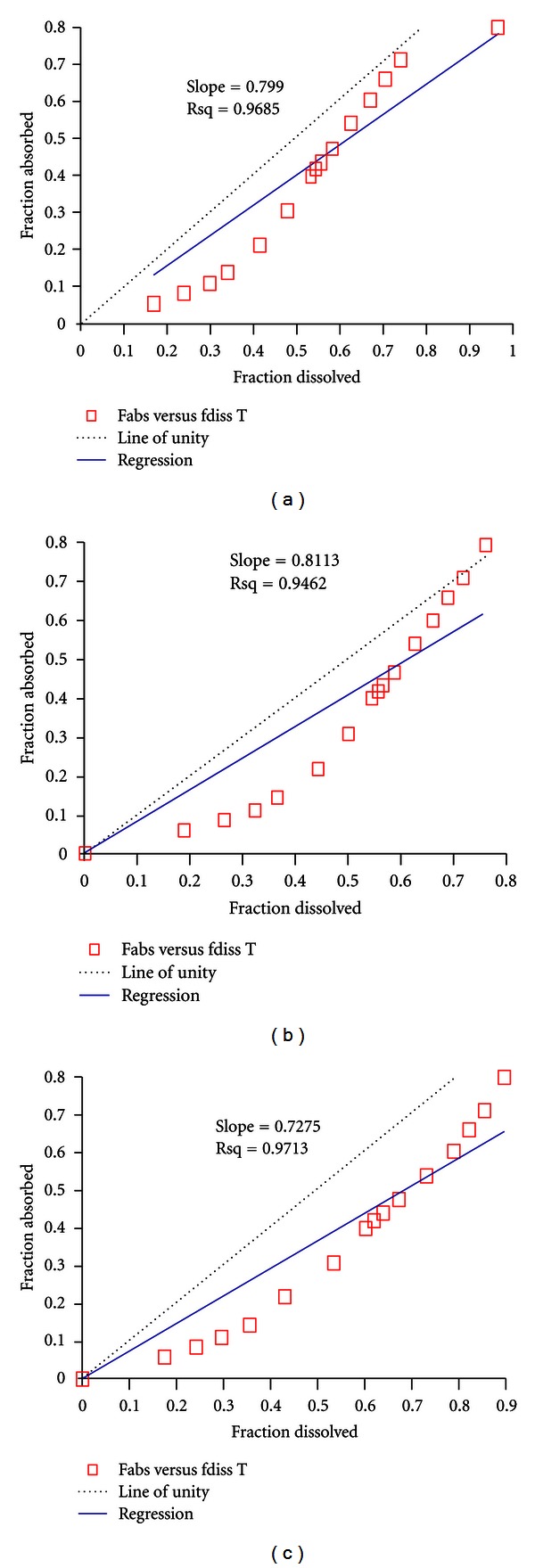
*In vivo-in vitro* correlation of fraction dissolve versus fraction of drug absorbed from alfuzosin 10** **mg MR tablets in (a) phosphate buffer (pH 6.8), (b) 0.1 N HCl (pH 1.2), and (c) acetate buffer (pH 4.7).

The linear plasma concentration (mean ± SD) versus time curves of 2 alfuzosin formulations applied to 35 subjects under fasting conditions are given in Figure 2. The mean *C*_max⁡_ (mean ± SD ng/mL) of test and reference formulation were 10.212 ± 2.23 and 11.422 ± 2.23, respectively, under fasting conditions. The mean AUCT (mean ± SD ng/mL ∗ hr) of test and reference were 196.172 ± 12.01 and 193.172 ± 12.01 while mean AUCI (mean ± SD ng/mL ∗ hr) of test and reference formulations were 211.468 ± 13.51 and 196.468 ± 13.51, respectively. The mean *T*_max⁡_ (mean ± SD h), *K*_el_ (mean ± SD h^−1^) and *t*_half_ (mean ± SD h) of test and reference formulations of alfuzosin were 6.00 ± 0.49 and 6.00 ± 0.69, 0.28 ± 0.1 and 0.27 ± 0.1, and 7.89 ± 0.87 and 7.89 ± 0.87, respectively, under fasting conditions. The least square mean of the primary PK parameters calculated from the ANOVA, the ratio and 95% confidence interval of primary PK parameters *C*_max⁡_, AUCT (AUClast), and AUCI (AUC total) assuming equal variance between the groups were 109% (99.03% to 122.78%), 104% (92.94% to 116.71%), and 110% (98.17% to 124.01%), respectively, under fasting conditions (Tables 4 and 5). The mean recovery of impurities A, B, D, and E (mean ± RSD) were 83.19 ± 1.89, 87.86 ± 3.26, 102.56 ± 0.86, and 87.97 ± 4.35, respectively, (Table S2 in Supplementary Material). 

### 3.5. Bioanalytical Method Validation Report in Human Plasma

**Assay Validation**

The assay procedure was validated by analyzing three single standard curve sets per day for a total of three days. The standard curve concentrations range from 0.050 to 30.0 ng/mL. On each day of validation, five sets of QC samples at 0.1500 ng/mL (low), 2.000 ng/mL (medium), and 25.00 ng/mL (high) were assayed for a total of twelve sets of QC samples for all six days. The QC concentration levels were selected to represent the full calibration range. An integrator was used to determine the chromatographic peak heights of alfuzosin and internal standard. The peak height ratios of alfuzosin to internal standard were used for the calculation of unknown concentrations.

### 3.6. IVIVC Study

The graph of fraction of drug dissolved *in vitro* versus fraction of drug absorb *in vivo* along with correlation coefficients at each dissolution media was separately constructed (Figures 2(a)–2(c)). The prediction error of the *C*_max⁡_ and AUC of the correlation were found to be −2.62% and 22.02% at pH 1.2 of dissolution media, −2.32% and 17.68% at pH 6.8 of dissolution media, and −2.38% and 18.74 at pH 4.7 of dissolution media, respectively (Table 6). 

## 4. Discussion

To develop alfuzosin 10 mg extended release tablets, active pharmaceutical ingredient (API) was characterized to meet the FDA regulation and showed comparative physicochemical and spectral characteristics with respect to the reference. The structural elucidation of finished test formulation was carried out by using IR, NMR (both proton and carbon), MASS, DSC, and XRD. The prominent IR peak for aromatic amine (N–H stretching), aromatic (=C–H stretching), secondary amide (C=O and C=H stretching) between test, and reference were similar, which confirmed the structural similarity of functional groups between test and reference drug. The proton NMR spectra between test and reference showed prominent peak at 11.9 ppm, 8.89 ppm, 7.7 ppm, and 3.34 ppm indicating similar chemical environment which was further confirmed by 13C NMR. The chemical shift of proton and carbon NMR between two drugs were similar and hence the structure of these two molecules was considered to be similar. Furthermore, molecular weight of both test and reference was 390 (Tables  S12–S15) from MASS spectrum for test and reference. Similar single pure peak at 339.33°C was obtained from the DSC of test and reference drug. The diffraction pattern between two drugs had similar 2*θ* angle peak which further confirmed the similar structure of the molecule between two dosage forms. Finally elemental analysis of test and reference showed no change in C, H, and N contents of the drugs implied the requirement as per compendial limit. These studies showed comparable spectral characteristics which signified sameness of test and reference dosage forms.

 A compatibility study of the drug with the selected impurities was conducted for the period of 30 days at 50°C. The analysis of the relative impurity was observed to find out the incompatibility. The selected inactive ingredients were compatible with the study drugs and hence can be used for the development of the formulation since the content of the impurities was within the quality limit (<0.11%).

The generic alfuzosin 10 mg ER tablets were prepared by hot-melt granulation using mono- and diglycerides as a rate controlling membrane [37]. Six different formulations (Table S3 in Supplementary Material) were prepared at different label of lactose, mono- and diglycerides and evaluated *in vitro* hardness, friability, and dissolution rate, color, texture, diameter, and width of the tablets for optimization of formulation [38–40]. Afterwards ten formulations were processed based on the *in vitro* parameters as mentioned in the reference drug. A batch formulation of the drug was also generated for further evaluations in the commercial scale. Finally a pivotal batch (Table 1) of alfuzosin hydrochloride ER tablets 10 mg was planned to make 130,000 tablets. 

The finished products were tested as it was tested for the raw API. The recovery of the impurities and relative retention factor (Table S2 in Supplementary Material) were less than 2% which indicated that the tablets met regulatory requirement of stability at the finished product. The residual solvent content (of methanol, ethanol, diethyl ether, acetone, isopropyl alcohol, dichloromethane, ethyl acetate, tetrahydrofuran) in the finished product was less than 1% which further satisfied the compendial requirement [29, 41].

The physicochemical characteristics of the finished products like hardness, assay, dissolution, and friability all are in the limit of the USP (United State Pharmacopeia standard). The *in vitro* dissolution of the test drug after 20 h was 87% as compare to the innovator product which was 103% showing slow controlled release of the drug from the dosage forms. Alfuzosin was released from the matrix tablets by diffusion and erosion mechanisms in all of the formulations. Assay of finished tablets were between 80 to 120% and RSD was below 10% which indicated that the product was equivalent to USP reference standard (Table S3 in Supplementary Material).

HPLC method validation for estimation of alfuzosin in human plasma was adequate, since method did not show any matrix effects and the accuracy of the method was 103% at the highest calibration concentration. The recovery of the internal standard was about 68% (Table 3). The highest accuracy was also obtained from the diluted standard which was 96.9% (Table S11 in Supplementary Material), bench top stability and stock solution stability and freeze thaw stability (Table S11 in Supplementary Material), and room temperature stability (Table S11 in Supplementary Material) were also within acceptable regulatory limit which signifies the validity of the method.

The generic alfuzosin HCl 10 mg ER tablets was prepared using mono- and diglyceride as oppose to the innovator formulation containing HPMC and ethyl cellulose. The difference in the formulation between test and innovator drug is presented in Table 7. So it falls under major changes, and hence to get marketing approval product needs to show bioequivalent to the innovator product in therapeutic outcome. Hence a bioequivalence study was conducted as per USFDA regulatory guideline for the generic products under fasting conditions in a two way cross over design. Cross-over design was chosen to establish bioequivalence, since cross over design reduced variability by using same subject in two different periods where genetic changes were no longer a factor to increase variability of the drug and also reduced the bias of selecting the pharmacogenomic unequal subjects between test and reference drugs. Hence the cross over design requires minimum subjects to establish bioequivalence of the generic drug. The bias of selecting the subjects in the equivalence trial was further removed by using randomization schedule (data not shown). 

A single dose two period, 2 sequence, 2 treatment cross-over study with a minimum wash-out period of 7 days was carried out in fasting conditions for alfuzosin formulation (FDA Guidelines, 2005). In the present cross-over study, 35 and 42 subjects were dosed in each of the study period under fasting conditions. Subjects were restricted to toilet and heavy physical exercise for first 2 h following administration of the drug in order to maintain similar environment. Study schedule and sampling schedule following drug administration were followed as per protocol. All the subjects were supplied a standard meal that had 950 to 1000 Kcal (kilo calorie) at 4.00, 8.00, and 12.00 h after drug administration. Since food may affect the absorption and dissolution of the drug and which in turn increased the variability of the PK parameters. In order to avoid such circumstances food with uniform calorie content was supplied to each of the volunteers during study period. A test drug is considered to be pharmacokinetic equivalent in turn bioequivalence to a reference drug product if the 90% CI of the test and reference geometric mean ratios of the AUCs and *C*_max⁡_ fall within 0.80 to 1.25. 

All subjects were monitored for vital sign, blood pressure and pulse rate to document any adverse events. Scheduled blood sample was taken from each subject in order to measure the drug in blood at each time point so that a concentration versus time graph can be constructed to describe rate and extend of absorption of the drug.

A HPLC method was validated to determine the concentration of the drug at each scheduled time points. The reproducibility of the method was tested using various trial batches. The linearity range (0.050 to 30.0 ng/mL) was fixed based upon the results of 6 trial batches. The heterosedasticity of various concentrations was eliminated by using appropriate weighing factor to construct the linear calibration curve. The maximum regression coefficient was fixed to greater than 0.9. The validity of the method was tested by studying matrix effects, dilution integrity stock solution stability at various temperatures (Table S11 in Supplementary Material), ruggedness, %recovery of the drug as well as internal standard, intra- and interday precession and accuracy (Table 3). The stability of the drug was tested against room temperature, freezing temperature, at bench top, reinjection, short-term and long-term stability of the drug (Table S11 in Supplementary Material). 

**Figure 3:**
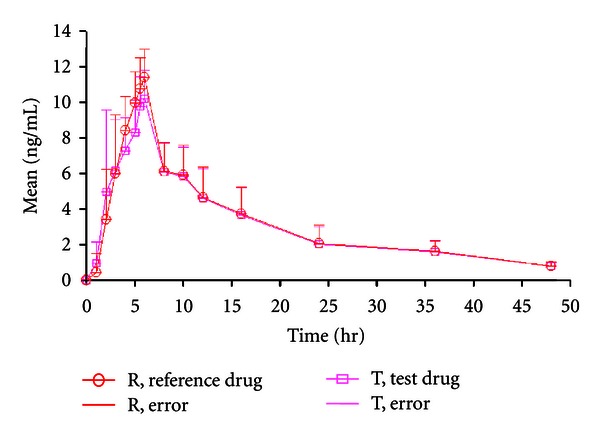
Linear mean plasma alfuzosin concentration (mean ± SD ng/mL) versus time (h) graph under fasting conditions (*n* = 35, R = reference formulation, T = test formulation).

The descriptive statistics of the individual subject concentration at each time points was used to construct a linear mean graph (Figure 3) and the significance of the linear mean graph was to compare PK profile between test and reference alfuzosin to conclude bioequivalence without statistical testing. In addition, semilog graph was constructed to show the parametric distribution of alfuzosin content in blood plasma and population therapeutic effects (Table 8).

The individual subject's PK parameters and descriptive statistics of the untransformed PK parameters (Tables 4 and 5) were tabulated for visual comparison between test and reference under fasting conditions before statistical analysis. Log transformation was done to bring the nonparametric data to parametric forms so that two one sided test and ANOVA can able to estimate the ratio and 90% confidence interval. Further sequence, period, and treatment effects were also calculated by the help of generalized linear model in Proc GLM in SAS v 9.2. In the present model sequence effects were tested at 10% level of significance whereas other effects were tested at 5% level of significance. In addition, subject nested within sequence as an error term used during modeling, were not shown in the data.

Significance of primary PK parameters was to draw conclusion whether a generic product can be given marketing authorization within the limit of efficacy safety and tolerability of an innovator drug. In the present study single dose pharmacokinetics study is similar to steady-state pharmacokinetics and healthy subjects' pharmacokinetics is proportional to the patients' pharmacokinetics. American Pharmaceutical Association (2003) has approved drug therapeutic equivalence information (2003). Hence pharmacokinetics evaluation is critical to establish therapeutic equivalence in turn establish bioequivalence. Therefore a single dose pharmacokinetics study was conducted to establish bioequivalence. The significance of all primary PK parameters are to access the rate and extend of drug absorption which corresponds to onset of action (*C*_max⁡_, rate of drug absorption) and duration of action (AUCT and AUCI) that is, total amount of drug absorbed. If the variability of these three parameters between test and reference are very narrow that is, about within ±20%, there will not be any chance to under action (sub therapeutic effects) or over action (adverse reaction). So, statistical equivalence of *C*_max⁡_, AUCT and AUCI between test and reference drug would bring a substituted generic which can switch over innovator. The primary PK parameters from this study, at 90% CIs were within the predefined bioequivalence criteria of 80 to 125% for the study under fasting conditions (Table 8). The PK study results revealed that the two formulations of alfuzosin were similar in PK characteristics among these healthy Indian subjects under fasting conditions. The 90% confidence intervals of log transformed PK metrics for the ratio of *C*_max⁡_, AUCT and AUCI were 99.03% to 122.78%, 92.94% to 116.71%, and 98.17% to 124.01% funder fasting conditions (Table 8) and met alfuzosin bioequivalence criteria as per EMEA regulation (FDA, guideline of orally administered drug's bioequivalence study).

Furthermore, the mean *t*_half_ (7.89 h) and *T*_max⁡_ (6.00 h) obtained from the test drug was comparable with reference drug's *t*_half_ (7.89 h) and *T*_max⁡_ (6.00 h) (Tables 4 and 5). The safety and tolerability of both the formulations was comparable. Adverse events were assessed for severity and relationship to the treatments throughout the study. Alfuzosin was well tolerated by all the volunteers with no clinically significant adverse events (AEs) such as increased neutrophil count, vomiting abdominal blotting and headache in the healthy subject. Since safety of the test formulation was better than the reference in the study, proposed changes in the formulation are acceptable and inactive ingredients used for the development of the formulations were safe. The inactive ingredients of the proposed generic formulation can be further changed to cheaper ingredients to bring down the production cost of the generic product and hence health care cost which warrants further research works. 

 A *in vivo in vitro* correlation (IVIVC) was tried to establish in order to select suitable *in vitro* dissolution media. To establish reasonable correlation, IVIVC link model was used which support linear regression only. The nonlinearity of observed values was adjusted with the variation by the link mode to generate level-A correlation. Since only linear correlation is accepted for the waiver approval, hence other nonlinear regression was not applied for better correlation. Based on the IVIVC analysis acetate buffer offered highest level A correlation of 0.97 (Figure 2(c)) and hence selected as a compendial media for future waiver of higher strength of same formulation without undergoing costly BE study.

## 5. Conclusion

The drug excipients compatibility studies were conducted and found satisfactory for intended purpose. The rate controlling polymer mono- and diglycerides had no significant impact on the impurity profile of the drug. Hence these polymers were considered as safe for the development of the extended release formulation as a rate controlling membrane. Since assay results of formulations and impurities were found within the acceptance criteria, the HPLC method was precise for quantification of impurities in alfuzosin hydrochloride API. *In vivo* study concluded that the test product is bioequivalence to the reference product in fasting conditions. The significant correlation between *in vitro* and *in vivo* parameters indicated that the IVIVC was excellent in predicting AUCT, but not acceptable in predicting *C*_max⁡_ because of high variability observed in maximum plasma concentrations among the healthy volunteers which is probably due to first pass metabolism. It is also observed that the least prediction errors of AUCT was −2.32 at pH 6.8, hence phosphate buffer pH 6.8 can be consider as a discriminative media for *in vitro* dissolution study. In final conclusion, the results suggested that the scale-up of highly permeable and highly soluble drugs did not significantly affect either *in vitro* dissolution or *in vivo* performance. It was further concluded based on the *in vivo* study that the test product was bioequivalence to the reference product in the present study.

## Supplementary Material

Details of formulation, process specification, testing procedure of API and impurities as well as characterization of Alfuzosin are reported in the supplementary document. In process specification and testing procedure fixed for pilot and pivotal formulation development and scale up are presented in Table S1. Table S2 presents relative retention time (in h) of the impurities of the alfuzosin by HPLC. Table S3 shows ratio of rate controlling membrane used for formulation development to evaluate the performance of the rate controlling agent in the drug release rate in pilot formulation whereas Table S4 presents result of in process parameters testing for pivotal and commercial production batch of alfuzosin 10 mg extended release formulation. Table S5 provides percentage of controlled drug release (% CDR) at each time points of 12 reference tablets UROXATRAL 10 mg extended release tablets at 0.1 N HCl (pH 1.2). Table S6 provides % CDR of at each time points of 12 test tablets of final alfuzosin 10 mg extended release tablets formulation at 0.1N HCl (pH 1.2). Similarly, Table S7 details % CDR of at each time points of 12 reference tablets Uroxatral (alfuzosin 10 mg) extended release tablets formulation at pH 4.7 and Table S8 narrates % CDR of at each time points of 12 test tablets of final alfuzosin 10 mg extended release tablets formulation batch at pH 4.7, while Table reports S9 % CDR of at each time points of 12 reference tablets Uroxatral (alfuzosin 10 mg) extended release tablets formulation at pH 6.8 and Table S10 mentions % CDR of at each time points of 12 reference tablets (alfuzosin 10 mg) extended release tablets formulation at pH 6.8. Table S11 has given results on Freeze and Thaw Stability of QC samples of Alfuzosin in Human Plasma (1 to 4 and 6 Cycles). IR peak assignment for both test and reference alfuzosin are reported in Table S12. Table S13 shows Proton NMR peak assignment and Table S14 for 13C NMR Chemical shift Assignment of alfuzosin test and standard drug. Mass spectrometric analysis reports are summarized in Table S15. Figure S1 has mentioned all steps for large scale manufacturing process of Alfuzosin and its formulation.

## Figures and Tables

**Table 1:** Composition of different scale up formulation and respective batch size.

Ingredients	*Spec	Placebo batch		Pivotal batch		Commercial production batch	
		RowSpanEmpty	Mg/tablets	Kg/batch	Mg/tablets	Kg/Batch	Mg/tablets	Kg/batch
Intragranular							
Alfuzosin HCl				10.000	0.5	10.000	1.300
Lactose monohydrate	NF	106.000	5.026*	106.000	5.3	106.000	13.780
Mono- and dig1ycerides (Imwitor 900)	NF	100.000	4.334	100.000	5.0	100.000	13.000
Extragranular							
Colloidal silicon dioxide (Aerosil 200)	NF	4.500	0.196	4.500	0.225	4.500	0.585
Magnesium stearate	NF	2.500	0.108	2.500	0.125	2.500	0.325
Talc (Luzenac)	USP	2.000	0.086	2.000	0.10	2.000	0.260

Total		215.000	9.750	225.000	11.25	225.000	29.250

*Specification.

**Table 2:** Cumulative % drug release of the pilot batches in 0.1 N HCl.

Time (h)	Reference	Alfuzosin hydrochloride ER tablets 10 mg (% drug release) (*n* = 6)					
		RowSpanEmpty	USP dissolution apparatus II (paddel) rpm 100; temperature 37°C ± 5°C					
Batch	25518	F005	F010	F015	F021	F022	F025 (optimal formulation)
1	17.80	22.32	22.39	20.32	19.66	25.45	19.39
2	24.71	31.11	32.03	31.07	27.71	33.06	28.27
3	30.67	36.98	38.69	40.23	32.87	48.19	34.61
4	36.33	41.92	45.10	44.70	37.62	55.60	40.44
8	54.27	56.80	61.75	63.25	52.92	71.25	57.19
12	70.04	68.77	74.83	77.81	64.55	79.42	68.70
16	85.42	78.48	86.96	88.92	74.52	89.92	83.36
24	98.05	92.89	100.00	102.23	86.90	104.09	96.99

**Table 3:** Intraday precision and accuracy for QC samples, LLOQ, and ULOQ for alfuzosin in human plasma (EDTA).

Batch run	HQC	MQC	LQC	LLOQ	ULOQ
	(25 ng/mL)	(2 ng/mL)	(0.150 ng/mL)	(0.050 ng/mL)	(30 ng/mL)
Mean	25.94	2.111	0.1543	0.04632	31.36
SD (*n* − 1)	0.7114	0.06474	0.01073	0.001933	0.4354
Precision	2.7	3.1	7	4.2	1.4
Accuracy (%)	104	106	103	92.6	105
BIAS (%)	3.8	5.6	2.9	−7.4	4.5
*n *	6	6	6	6	6
Overall mean	25.88	2.069	0.1554	0.05092	30.97
SD (*n* − 1)	1.415	0.1162	0.009395	0.004951	1.578
Precision	5.5	5.6	6	9.7	8.1
Accuracy (%)	104	103	104	102	103
BIAS (%)	3.5	3.5	3.6	3.8	3.2
*n *	18	18	18	18	18
Recovery	Internal standards was 68% and analyte was 79%				

**Table 4:** Descriptive pharmacokinetics properties of the reference treatment under fasting conditions (*n* = 35).

Statistics	*K*_el_	*T*_half_	*C*_max⁡_	*T*_max⁡_	AUCT	AUCI	AUC_Exp
	RowSpanEmpty	(h^−1^)	(h)	(ng/mL)	(h)	(ng/mL ∗ h)		(ng/mL ∗ h)
Mean	0.2679	7.89	10.2117	6.00	193.1723	196.4683	8.80
SD	0.10	0.47	2.23	0.69	12.0139	13.51	2.66
Min	0.1488	6.46	3.2445	0.33	119.1388	130.8190	4.92
Median	0.2191	7.16	8.3611	5.5	187.5498	180.5020	8.92
Max	0.4753	9.66	15.6322	8.00	250.6483	198.6327	15.53
CV%	37.13	30.13	26.89	50.55	36.22	37.04	30.26
Geometric mean	0.2524	7.75	10.9862	6.86	190.2896	193.2267	8.43
Harmonic mean	0.2397	7.59	10.6595	6.77	194.6518	199.2482	8.08

**Table 5:** Descriptive pharmacokinetics properties of the test treatment under fasting conditions (*n* = 35).

Statistics	*K*_el_	*T*_half_	*C*_max⁡_	*T*_max⁡_	AUCT	AUCI	AUC_Exp
	RowSpanEmpty	(h^−1^)	(h)	(ng/mL)	(h)	(ng/mL ∗ h)		(ng/mL ∗ h)
Mean	0.2779	7.89	11.4224	6.00	196.1723	211.4683	8.80
SD	0.10	0.87	2.23	0.49	12.0139	13.51	2.66
Min	0.1488	6.46	6.2445	4.33	119.1388	186.8190	4.92
Median	0.2191	7.16	9.3611	6.00	177.5498	179.5020	8.92
Max	0.4753	9.66	15.6322	8.00	250.6483	258.6327	15.53
CV%	37.13	30.13	26.89	50.55	36.22	37.04	30.26
Geometric mean	0.2524	7.75	11.9862	6.86	198.2896	213.2267	8.43
Harmonic mean	0.2397	7.59	11.6595	6.77	201.6518	216.2482	8.08

**Table 6:** *In vitro-in vitro* correlation table at different dissolution media to select discriminative dissolution media for *in vitro* drug release.

Dissolution medium pH	PK parameters	Predicted	Observed	% PE	Ratio
1.2	AUClast (ng/mL ∗ h)	61.70	63.35	−2.62	0.97
	*C*_max⁡_ (ng/mL)	1.33	1.09	22.02	1.22

4.7	AUClast (ng/mL ∗ h)	61.85	63.35	−2.38	0.98
	*C*_max⁡_ (ng/mL)	1.29	1.09	18.74	1.19

6.8	AUClast (ng/mL ∗ h)	61.88	63.35	−2.32	0.98
	*C*_max⁡_ (ng/mL)	1.28	1.09	17.68	1.18

**Table 7:** Functions of excipients in reference listed drug (RLD) and developed generic product.

Reference listed drug	Proposed developing generic drug	Function
Microcrystalline cellulose, NF	Lactose monohydrate, NF	Filler
Mannitol, USP		Filler
Hydroxypropyl methylcellulose, USP	Mono- and diglycerides, NF	Rate controlling polymer
Ethylcellulose, NF		Polymer
Colloidal silicon dioxide, NF	Colloidal silicon dioxide, NF	Glidant
Magnesium stearate	Magnisium stearate	Lubricant
Hydrogenated castor oil, NF	Talc	Lubricant
Povidone		Solubilizer
Yellow iron oxide		Colourant

**Table 8:** Log transformed PK parameters.

Pharmacokinetic parameters	Test geometric mean	Reference geometric mean	Test/reference ratio	90% confidence interval for test versus reference	Power	Intrasubject CV
Ln_*C*_max⁡_	11.986	10.986	109%	99.03% to 122.78%	0.998	25.16%
Ln_AUCT	198.289	190.289	104%	92.94% to 116.71%	1.000	27.44%
Ln_AUCI	213.227	193.227	110%	98.17% to 124.01%	1.000	27.02%

## Abbreviations

AARAS: Selective alpha-adrenergic receptor antagonists
BCS: Biopharmaceutical classification system
BPH: Benign prostatic hyperplasia
ER: Extended release
K3EDTA: Tripotassium ethylene diaminetetra acetic acid
LUTS: Lower urinary tract syndromes
NF: National formularies
R: Reference formulation
RH: Relative humidity
SPE: Solid Phase extraction
T: Test formulation
USP: United State pharmacopoeia
ALT: Alanine transaminase
AST: Aspartate aminotransferase
RIA: Radioactive immunoassay.
