# Supplementary material for: In Vitro-In Vivo Correlation Evaluation of Generic Alfuzosin Modified Release Tablets
Source: ISRN Toxicol. 2012 Nov 20;2012:813836. doi: 10.5402/2012/813836 (PMC3671720; doi:10.5402/2012/813836)
Supplement: Supplementary file 1 — Details of formulation, process specification, testing procedure of API and impurities as well as characterization of Alfuzosin are reported in the supplementary document. In process specification and testing procedure fixed for pilot and pivotal formulation development and scale up are presented in Table S1. Table S2 presents relative retention time (in h) of the impurities of the alfuzosin by HPLC. Table S3 shows ratio of rate controlling membrane used for formulation development to evaluate the performance of the rate controlling agent in the drug release rate in pilot formulation whereas Table S4 presents result of in process parameters testing for pivotal and commercial production batch of alfuzosin 10 mg extended release formulation. Table S5 provides percentage of controlled drug release (% CDR) at each time points of 12 reference tablets UROXATRAL 10 mg extended release tablets at 0.1 N HCl (pH 1.2). Table S6 provides % CDR of at each time points of 12 test tablets of final alfuzosin 10 mg extended release tablets formulation at 0.1N HCl (pH 1.2). Similarly, Table S7 details % CDR of at each time points of 12 reference tablets Uroxatral (alfuzosin 10 mg) extended release tablets formulation at pH 4.7 and Table S8 narrates % CDR of at each time points of 12 test tablets of final alfuzosin 10 mg extended release tablets formulation batch at pH 4.7, while Table reports S9 % CDR of at each time points of 12 reference tablets Uroxatral (alfuzosin 10 mg) extended release tablets formulation at pH 6.8 and Table S10 mentions % CDR of at each time points of 12 reference tablets (alfuzosin 10 mg) extended release tablets formulation at pH 6.8. Table S11 has given results on Freeze and Thaw Stability of QC samples of Alfuzosin in Human Plasma (1 to 4 and 6 Cycles). IR peak assignment for both test and reference alfuzosin are reported in Table S12. Table S13 shows Proton NMR peak assignment and Table S14 for 13C NMR Chemical shift Assignment of alfuzosin test and standa [file 813836.f1.docx]

**Table S1: In process specification and testing procedure fixed for pilot and pivotal formulation development and scale up.**

| **Sr. No** | **Test** | **Specification** |
| --- | --- | --- |
| **1** | **Description** | White to off-white, round, beveled biconvex, uncoated tablets, both are plain. |
|  | **Identification by uv** | UV absorption spectrum of sample preparation exhibits  maxima and minima, which are at the same wavelengths as  that of standard preparation, as obtained in the assay. |
|  | **Blend uniformity by UV** | (i) Mean of all results is 90.0 % to 110.0 % of target assay.  (ii) RSD of all individual results: Not more than 5.0 % |
|  | **Assay by UV** | Each 225 mg granular powder contains  Not less than 9.50 mg and not more than 11.00 mg  (95.0 % to 110.0 % of labeled amount) |
| 2 | **Average weight of tablets (Weight of 20 tablets/20)** | 225.0 mg ± 3.0 %w/w  (218.0 mg to 232.0 mg) |
| 3 | **Individual weight variation** | Actual average weight (mg) ± 5.0 %w/w. NMT two of tablets should deviate by more than 5.0 % w/w and none should deviates by more than 10.0 % w/w of actual average weight. |
| 4 | **Resistance to crushing (Hardness)** | Target: 4.5 kp  Limit: 3.0 kp to 6.5 kp) |
| 5 | **Thickness** | 4.5 mrn + 0.5 mm  (4.0 mm to 5.0 mm) |
| 6 | **Friability** | NMT 1.0%w/w |
| 7 | **Diameter*** | 7.9 mm ± 0.2 mm (7.7 mm to 8.1 mm) |

**Table S2: Relative retention time (in h) of the impurities of the alfuzosin**

| Name | Relative retention  time (Approx.) | RRF (relative retention factor) | LOQ (%) limit of quantitation |
| --- | --- | --- | --- |
| Impurity A | 1.20 | 0.765 | 0.026 |
| Impurity B | 0.53 | 0.664 | 0.026 |
| Impurity D | 0.45 | 0.829 | 0.025 |
| Impurity E | 0.47 | 0.882 | 0.025 |
| Alfuzosin HCL | 1.00 |  |  |

**Table S3: Ratio of rate controlling membrane used for formulation development to evaluate the performance of the rate controlling agent in the drug release rate in pilot formulation**

| Experiment No | Mono and di glycerides  Level (%) | Lactose Monohydrate Level (%) | Tablet Wt  Mg | Hardness  (Kp) | Friability  (%) |
| --- | --- | --- | --- | --- | --- |
| F005 | 30 | 61 | 200 | 4.68 | 0.92 |
| F010 | 35 | 56 | 200 | 4.45 | 0.83 |
| F015 | 30 | 60 | 200 | 4.56 | 0.74 |
| F021 | 46.7 | 45.1 | 225 | 4.6 | 0.84 |
| F022 | 35 | 51 | 200 | 4.36 | 0.63 |
| F025 | 44.4 | 47.1 | 225 | 4.5 | 0.63 |

**Table S4 : Result of in process parameters testing for pivotal and commercial production batch of alfuzosin 10 mg extended release formulation**

| *In-process Parameter* | *Exhibit Batch* | *Intended Commercial Production*  *Batches* |
| --- | --- | --- |
| Description | White to off-white, round, beveled biconvex, uncoated tablets, both are plain. | White to off-white, round, beveled biconvex, uncoated tablets, both are plain. |
| Identification  (By UV) | UV absorption spectrum of sample  Preparation exhibits maxima and minima, which are at the same wavelengths as that of standard preparation, as obtained in the assay. | UV absorption spectrum of sample  Preparation exhibits maxima and minima, which are at the same wavelengths as that of standard preparation, as obtained in the assay. |
| Blend uniformity  (By UV) | (1)103.5% (2) 101.8% (3) 101.5%  (4) 100.8% (5)99.5% (6) 98.6%  (7) 100.4% (8) 98.7% (9) 98.2%  (10) 98.7%  Mean: 100.2%  Min. : 98.2%  Max. : 103.5%  RSD 1.7% | (1)103.5% (2) 101.8% (3) 101.5%  (4) 100.8% (5)99.5% (6) 98.6%  (7) 100.4% (8) 98.7% (9) 98.2%  (10)98.7%  Mean: 100.2%  Min. : 98.2%  Max. : 103.5%  RSD 1.7% |
| ASSAY (BY UV) | Each 225 mg of granular ,powder contains: Alfuzosin hydrochloride  10.08 mg (100.8%) | Each 225 mg of granular ,powder contains: Alfuzosin hydrochloride  10.1 mg (101%) |
| Average weight of tablets (Weight of 20 tablets/20) | 225.0 mg ± 3.0 %w/w  (218.0 mg to 232.0 mg) | 225.0 mg ± 3.0 %w/w  (218.0 mg to 232.0 mg) |
| Individual weight variation | Actual average weight (mg) ± 5.0  %w/w. NMT two of tablets should deviate by more than 5.0 % w/w and  none should deviates by more than  10.0 % w/w of actual average weight. | Actual average weight (mg) ± 5.0  %w/w. NMT two of tablets should deviate by more than 5.0 % w/w and none should deviates by more than 10.0 % w/w of actual average weight. |
| Resistance to crushing (Hardness) | Target: 4.5 kp  Limit: 3.0 kp to 6.5 kp) | Target: 4.5 kp  (Limit: 3.0kp to 6.5 kp) |
| Thickness | 4.5 mrn + 0.5 mm  (4.0 mm to 5.0 mm) | 4.50 mm + 0.50 mm  (4.00 mm to 5.00 mm) |
| Friability | NMT 1.0%w/w | NMT 1.0%w/w |
| Diameter* | 7.9 mm ± 0.2 mm (7.7 mm to 8.1 mm) | 7.90 mm± 0.20mm (7.70 to 8.10 mm) |

**Table S5: % CDR at each time points of 12 reference tablets UROXATRAL 10 mg extended release tablets at 0.1 N HCL (pH 1.2)**

| Tablet | Time in hours (% cumulative drug release ) | | | | | | | | | | |
| --- | --- | --- | --- | --- | --- | --- | --- | --- | --- | --- | --- |
| No | 1 | 2 | 3 | 4 | 6 | 8 | 10 | 12 | 16 | 20 | 24 |
| 1 | 16.1 | 22.2 | 28.3 | 33.3 | 41.3 | 48.6 | 55.8 | 64 | 89.7 | 95 | 97.3 |
| 2 | 17.6 | 25.2 | 30.6 | 36 | 41.4 | 49.2 | 55.2 | 62.4 | 85.8 | 89 | 92.7 |
| 3 | 16.3 | 23.7 | 30 | 35.5 | 45.2 | 53.6 | 62.5 | 69.9 | 88.1 | 95.8 | 99.4 |
| 4 | 15.6 | 23 | 29.8 | 36.1 | 46.1 | 58.4 | 71.5 | 82.2 | 96.4 | 102.4 | 102.2 |
| 5 | 14.9 | 23.6 | 29.1 | 34 | 40.3 | 47.6 | 57.4 | 64.7 | 79.2 | 91.8 | 97.2 |
| 6 | 16.8 | 23.8 | 30.3 | 33.7 | 39.6 | 48.2 | 58.2 | 73.2 | 83.6 | 93.5 | 99.8 |
| 7 | 17.6 | 24.9 | 30.1 | 34.7 | 41.3 | 50.3 | 56 | 64.6 | 80.1 | 89.5 | 88.6 |
| 8 | 16.6 | 24 | 27.4 | 34.2 | 43 | 50.2 | 57.8 | 65.5 | 84.6 | 94.9 | 93.8 |
| 9 | 17.7 | 24.6 | 32.4 | 39.8 | 50.5 | 60.5 | 69.6 | 79.9 | 92.4 | 102 | 96.4 |
| 10 | 16.9 | 24.5 | 30.9 | 37.3 | 45.6 | 53.4 | 62.2 | 68.2 | 85 | 96.4 | 91.7 |
| 11 | 16 | 24 | 29.6 | 33.5 | 43 | 51 | 57.9 | 66.5 | 83 | 93.3 | 91.6 |
| 12 | 19.2 | 26.2 | 31.7 | 36.6 | 43.8 | 51.9 | 58.6 | 66.5 | 78.6 | 88 | 89.1 |
| Min | 14.9 | 22.2 | 27.4 | 33.3 | 39.6 | 47.6 | 55.2 | 62.4 | 78.6 | 88 | 88.6 |
| Max | 19.2 | 26.2 | 32.4 | 39.8 | 50.5 | 60.5 | 71.5 | 82.2 | 96.4 | 102.4 | 102.2 |
| Mean | 16.8 | 24.1 | 30 | 35.4 | 43.4 | 51.9 | 60.2 | 69 | 85.5 | 94.3 | 95 |
| RSD | 6.8 | 4.3 | 4.6 | 5.4 | 7.1 | 7.7 | 8.9 | 9.2 | 6.3 | 4.9 | 4.6 |

**Table S6: % CDR of at each time points of 12 test tablets of final alfuzosin 10 mg extended release tablets formulation at 0.1N HCL (pH 1.2)**

| Tablet | Time in hours (% cumulative drug release ) | | | | | | | | | | |
| --- | --- | --- | --- | --- | --- | --- | --- | --- | --- | --- | --- |
| No | 1 | 2 | 3 | 4 | 6 | 8 | 10 | 12 | 16 | 20 | 24 |
| 1 | 18.3 | 25.8 | 32.2 | 36.3 | 45.8 | 50.2 | 54.8 | 58.5 | 65.5 | 72.4 | 75.9 |
| 2 | 20.1 | 27.3 | 33.2 | 37.5 | 45.4 | 52.3 | 57.2 | 61.2 | 69.5 | 73.8 | 77.6 |
| 3 | 18.9 | 27 | 33 | 37 | 44.8 | 50.8 | 56.2 | 60.6 | 68.6 | 74.7 | 78.5 |
| 4 | 18.6 | 26.9 | 32.5 | 38.5 | 44.4 | 50.8 | 55.3 | 59.4 | 68 | 72.3 | 76.4 |
| 5 | 17.8 | 26 | 31.4 | 34.9 | 42.6 | 48.2 | 52.8 | 56.8 | 64.8 | 70.2 | 75 |
| 6 | 17.6 | 25.7 | 31 | 35.1 | 42.9 | 48.4 | 53.2 | 57.4 | 64.7 | 70.6 | 74.6 |
| 7 | 19.1 | 26.7 | 32.4 | 37.6 | 45.2 | 51.3 | 56.9 | 60.5 | 65.2 | 70.3 | 75.5 |
| 8 | 18.5 | 26 | 31.9 | 35.9 | 44.1 | 49.5 | 54 | 58.1 | 65.2 | 71.9 | 75.7 |
| 9 | 19.4 | 26.7 | 31.9 | 36.5 | 44.3 | 49.7 | 54.6 | 59 | 65.4 | 72.2 | 75.5 |
| 10 | 19.2 | 26.9 | 32.4 | 36.7 | 44.1 | 49.7 | 54.5 | 58.9 | 66.1 | 71.9 | 75.8 |
| 11 | 18.8 | 26.8 | 32.3 | 36.7 | 44.6 | 49.8 | 53.8 | 59.1 | 66.7 | 72.8 | 77.2 |
| 12 | 19.1 | 27 | 32.8 | 36.8 | 44.1 | 50.5 | 54.7 | 60 | 66.6 | 72.3 | 77.8 |
| Min | 17.6 | 25.7 | 31 | 34.9 | 42.6 | 48.2 | 52.8 | 57.4 | 64.7 | 70.2 | 74.6 |
| Max | 20.1 | 27.3 | 33.2 | 38.5 | 45.8 | 52.3 | 57.2 | 61.2 | 69.5 | 74.7 | 78.5 |
| Mean | 18.8 | 26.6 | 32.3 | 36.6 | 44.4 | 50.1 | 54.8 | 59.1 | 66.4 | 72.1 | 76.3 |
| RSD | 3.7 | 2 | 2 | 2.8 | 2.1 | 2.3 | 2.5 | 2.2 | 2.4 | 1.8 | 1.6 |

**Table S7: % CDR of at each time points of 12 reference tablets Uroxatral (alfuzosin 10mg) extended release tablets formulation at pH 4.7**

| Tablet | Time in h (% cumulative drug release ) | | | | | | | | | | |
| --- | --- | --- | --- | --- | --- | --- | --- | --- | --- | --- | --- |
| No | 1 | 2 | 3 | 4 | 6 | 8 | 10 | 12 | 16 | 20 | 24 |
| 1 | 19 | 25.1 | 29.6 | 35.8 | 39.7 | 54.4 | 58 | 65.5 | 78.8 | 87.7 | 90.7 |
| 2 | 18 | 25.1 | 29.6 | 35.8 | 40.3 | 54.4 | 58 | 65.5 | 77.6 | 87.8 | 90.2 |
| 3 | 18 | 25.2 | 28.1 | 35.3 | 43.2 | 52.9 | 63 | 69.7 | 78.2 | 85.5 | 89.9 |
| 4 | 18 | 22.8 | 29.2 | 35.3 | 43.1 | 52.8 | 63.2 | 66.5 | 78.6 | 85.6 | 89 |
| 5 | 16.8 | 22.9 | 30.1 | 35.2 | 43 | 52.7 | 59.6 | 66.3 | 78.6 | 84.8 | 89.2 |
| 6 | 16.9 | 24 | 29.9 | 36.4 | 46.2 | 51.8 | 59.5 | 66.7 | 78.8 | 84 | 88.1 |
| 7 | 17.5 | 23.7 | 31.2 | 36.5 | 45.2 | 55.1 | 58.2 | 65.4 | 76.8 | 85.1 | 89.8 |
| 8 | 17.5 | 23.3 | 30.4 | 33.7 | 43.6 | 52.4 | 58.4 | 70.4 | 83.2 | 85.5 | 89.8 |
| 9 | 17.5 | 22.3 | 27.6 | 36.3 | 42.1 | 53.1 | 62.6 | 69.3 | 81.9 | 84.4 | 89.2 |
| 10 | 16.7 | 25 | 29.8 | 36.1 | 42.9 | 53.7 | 62.7 | 66.2 | 79.8 | 86.4 | 91.1 |
| 11 | 16.7 | 25 | 30.1 | 35.3 | 42.7 | 53.6 | 62.8 | 68.3 | 78.3 | 85.8 | 91.1 |
| 12 | 19 | 24.6 | 31.1 | 34.7 | 44.7 | 55.2 | 59.3 | 67.9 | 76.8 | 85.3 | 90.3 |
| Min | 16.7 | 22.8 | 27.6 | 33.7 | 39.7 | 51.8 | 58 | 65.4 | 76.8 | 84 | 88.1 |
| Max | 19 | 25.2 | 31.2 | 36.5 | 46.2 | 55.2 | 63.2 | 70.4 | 83.2 | 87.8 | 91.1 |
| Mean | 17.6 | 24.1 | 29.7 | 35.5 | 43.1 | 53.5 | 60.4 | 67.3 | 79 | 85.7 | 89.9 |
| RSD | 4.6 | 4.4 | 3.6 | 2.3 | 4.3 | 2 | 3.6 | 2.6 | 2.4 | 1.4 | 1 |

**Table S8: % CDR of at each time points of 12 test tablets of final alfuzosin 10 mg extended release tablets formulation batch at pH 4.7**

| Tablet | Time in h (% cumulative drug release ) | | | | | | | | | | |
| --- | --- | --- | --- | --- | --- | --- | --- | --- | --- | --- | --- |
| No | 1 | 2 | 3 | 4 | 6 | 8 | 10 | 12 | 16 | 20 | 24 |
| 1 | 17.9 | 25.8 | 31.2 | 36.2 | 42.8 | 50.1 | 55.8 | 59.9 | 68.3 | 74 | 17.9 |
| 2 | 18.8 | 26.4 | 31.6 | 36.6 | 44.5 | 51.2 | 56.5 | 61.7 | 69.9 | 78.2 | 18.8 |
| 3 | 17.9 | 25.8 | 31.6 | 36.2 | 44.3 | 50.6 | 55.9 | 60.7 | 68.9 | 75.4 | 97.9 |
| 4 | 18.2 | 24.8 | 32.2 | 35.2 | 42.9 | 51 | 56 | 59.8 | 67.1 | 75 | 98.2 |
| 5 | 18.9 | 24.8 | 30.3 | 35.1 | 42.8 | 49.7 | 54.6 | 59.3 | 67.7 | 75 | 98.9 |
| 6 | 17.3 | 27.4 | 32.6 | 37.7 | 46.2 | 52.7 | 58.4 | 63.3 | 71.8 | 78.8 | 97.3 |
| 7 | 19.1 | 26.7 | 32.4 | 37.5 | 43.3 | 51.3 | 57 | 61.8 | 70.4 | 77.3 | 99.1 |
| 8 | 18.2 | 25.7 | 31.4 | 35.9 | 42.8 | 49.6 | 54.9 | 59.4 | 68.5 | 74.9 | 98.2 |
| 9 | 18.7 | 26.3 | 31.8 | 37.1 | 44.8 | 51.1 | 57.6 | 62.1 | 70.8 | 77.1 | 98.7 |
| 10 | 18.9 | 26.4 | 32.3 | 37.3 | 45.8 | 51.4 | 57.3 | 62.1 | 71 | 77.2 | 98.9 |
| 11 | 18.4 | 26.2 | 32.3 | 36.9 | 44.6 | 50.6 | 56.5 | 61.4 | 70 | 77.3 | 98.4 |
| 12 | 19.7 | 26.7 | 32.6 | 37.7 | 45.7 | 52.2 | 58.2 | 63.5 | 72.2 | 79.9 | 99.7 |
| Min | 17.3 | 24.8 | 30.3 | 35.1 | 42.8 | 49.6 | 54.6 | 59.3 | 67.1 | 74 | 97.3 |
| Max | 19.7 | 27.4 | 32.6 | 37.7 | 46.2 | 52.7 | 58.4 | 63.5 | 72.2 | 79.9 | 99.7 |
| Mean | 18.5 | 26.1 | 31.9 | 36.6 | 44.2 | 51 | 56.6 | 61.3 | 69.7 | 76.7 | 98.5 |
| RSD | 3.5 | 2.9 | 2.1 | 2.5 | 2.9 | 1.8 | 2.1 | 2.3 | 2.3 | 2.4 | 3.5 |

**Table S9: % CDR of at each time points of 12 reference tablets Uroxatral (alfuzosin 10mg) extended release tablets formulation at pH 6.8**

| Tablet | Time in h (% cumulative drug release ) | | | | | | | | | | |
| --- | --- | --- | --- | --- | --- | --- | --- | --- | --- | --- | --- |
| No | 1 | 2 | 3 | 4 | 6 | 8 | 10 | 12 | 16 | 20 | 24 |
| 1 | 18.7 | 21.5 | 27.4 | 32.1 | 38.8 | 45.7 | 52 | 56.9 | 70.6 | 80.3 | 87.2 |
| 2 | 18.5 | 20.4 | 24.8 | 28.9 | 35.8 | 41.5 | 46 | 51.1 | 64.8 | 73.7 | 79.8 |
| 3 | 17.6 | 20 | 25 | 29.5 | 38.9 | 46 | 52.3 | 57.9 | 72.5 | 82.2 | 86 |
| 4 | 19.3 | 22.4 | 29.5 | 34.1 | 41.4 | 49.5 | 54.4 | 60.6 | 74.1 | 82.7 | 86.2 |
| 5 | 16.9 | 19.8 | 25.4 | 28.7 | 35.1 | 41.1 | 45.7 | 51.2 | 64.7 | 73 | 78.8 |
| 6 | 18 | 20.6 | 25.2 | 29.2 | 35.4 | 41.6 | 46.3 | 53.2 | 66.7 | 77.7 | 85.2 |
| 7 | 15.7 | 20.4 | 25.5 | 30 | 34.6 | 40.5 | 47 | 52.4 | 62.4 | 71.6 | 79.3 |
| 8 | 14.9 | 21 | 25.7 | 29.9 | 35.1 | 41.6 | 47.9 | 53.9 | 65.1 | 74.9 | 81.1 |
| 9 | 15.3 | 21.4 | 26.3 | 32 | 39.8 | 47.4 | 55.1 | 62 | 77.9 | 87 | 90.2 |
| 10 | 14.9 | 23.4 | 25.1 | 29.4 | 35.4 | 41.1 | 46.7 | 53 | 623 | 72.6 | 81.1 |
| 11 | 16 | 23.4 | 28 | 32.5 | 38.3 | 45.2 | 50.4 | 55.4 | 65.5 | 74 | 80.3 |
| 12 | 15.5 | 21.4 | 25.5 | 29.7 | 35.7 | 41.7 | 47.2 | 52.8 | 63 | 73.8 | 81 |
| Min | 14.9 | 19.8 | 24.8 | 28.7 | 34.6 | 40.5 | 45.7 | 51.1 | 62.3 | 71.6 | 78.8 |
| Max | 19.3 | 23.4 | 28 | 34.1 | 41.4 | 49.5 | 55.1 | 62 | 77.9 | 87 | 90.2 |
| Mean | 16.8 | 21.3 | 26.1 | 30.5 | 37 | 43.6 | 49.3 | 55 | 67.5 | 77 | 83 |
| RSD | 9.5 | 5.7 | 5.5 | 5.7 | 6.1 | 6.9 | 6.9 | 6.5 | 7.5 | 6.4 | 4.5 |

**Table S10: % CDR of at each time points of 12 reference tablets (alfuzosin 10mg) extended release tablets formulation at pH 6.8**

| Tablet | Time in h (% cumulative drug release ) | | | | | | | | | | |
| --- | --- | --- | --- | --- | --- | --- | --- | --- | --- | --- | --- |
| No | 1 | 2 | 3 | 4 | 6 | 8 | 10 | 12 | 16 | 20 | 24 |
| 1 | 16.6 | 23.6 | 29.6 | 34.4 | 42.3 | 48.5 | 54.4 | 59.1 | 68.2 | 74.6 | 96.6 |
| 2 | 16.9 | 24 | 29.5 | 33.9 | 42 | 48.7 | 54 | 59.5 | 68.8 | 76.8 | 96.9 |
| 3 | 16.6 | 23.8 | 29 | 34.1 | 41.6 | 47.6 | 53.8 | 58 | 66.9 | 74.1 | 96.6 |
| 4 | 17.2 | 24.4 | 34.3 | 34.3 | 40.8 | 47.3 | 53.4 | 57.3 | 67 | 73 | 97.2 |
| 5 | 16.9 | 24 | 29.1 | 33.6 | 41.1 | 48.1 | 53.2 | 57.9 | 65.5 | 72.3 | 96.9 |
| 6 | 16.7 | 23.7 | 29 | 34.1 | 41.3 | 48.3 | 53.3 | 58.3 | 66.2 | 74.8 | 96.7 |
| 7 | 16.8 | 23.6 | 29.5 | 33.9 | 40.8 | 47.9 | 53.2 | 58.5 | 67.4 | 74.7 | 96.8 |
| 8 | 16.8 | 24.1 | 29.6 | 33.9 | 41.2 | 48.2 | 53.7 | 58.4 | 67.2 | 74.8 | 96.8 |
| 9 | 17.1 | 23.8 | 30 | 34 | 41.9 | 48.1 | 53.6 | 58.6 | 67.7 | 74.4 | 97.1 |
| 10 | 16.8 | 24.6 | 29.5 | 34 | 41.6 | 47.3 | 533 | 58.2 | 67.6 | 73.8 | 96.8 |
| 11 | 16.9 | 23.7 | 29.6 | 34 | 41.5 | 47.6 | 53.2 | 58.2 | 66.6 | 73.6 | 96.9 |
| 12 | 17 | 23.9 | 29.4 | 34.1 | 41.6 | 47.5 | 53.3 | 58 | 66.8 | 74.9 | 97 |
| Min | 16.6 | 23.6 | 29 | 33.6 | 40.8 | 47.3 | 53.2 | 57.3 | 65.5 | 72.3 | 96.6 |
| Max | 19.3 | 23.4 | 28 | 34.1 | 41.4 | 49.5 | 55.1 | 62 | 77.9 | 87 | 99.3 |
| Mean | 16.9 | 23.9 | 29.8 | 34 | 41.5 | 47.9 | 53.5 | 58.3 | 67.2 | 74.3 | 96.9 |
| RSD | 1.1 | 1.3 | 4.8 | 0.6 | 1.1 | 1 | 0.7 | 1 | 1.3 | 1.5 | 1.0 |

**Table S11: Freeze and Thaw Stability of QC samples of Alfuzosin in Human Plasma (I to 4 and 6 Cycles)**

| AFTER ONE CYCLE | HIGH  25ng/ml | MEDIUM  2 ng/mL | LOW  0.15ng /ml |
| --- | --- | --- | --- |
|  |  |  |  |
| MEAN | 25.47 | 2.049 | 0.1446 |
| %CHANGE | 1.9 | 2.5 | -3.6 |
| Room temperature after 25hrs |  |  |  |
| NOMINAL value ng/ml | 25 | 2 | 0.15 |
| %CHANGE | 1.7 | 3.2 | 5 |
| Refrigerator stability 4°C ±6°C after 71 Hours |  |  |  |
| NOMINAL value ng/ml | 25 | 2 | 0.15 |
| %CHANGE | -8.9 | -8.3 | -10.8 |
| Stock solution stability |  |  |  |
| Refrigerator () % change | 0.6 | .70 | 1.2 |
| Room temperature | 2.3 | 2.9 | 3.1 |

Table S12: IR peak assignment for both test and reference alfuzosin

| Frequency (1/cm) | | Assignment |
| --- | --- | --- |
| Reference | Test |  |
| 3371.57 | 3369.64 | -N-H aromatic stretching (primary amine) |
| 3138.18 | 3068.75,3049.46 | *=C-H aromatic stretching* |
| 2935.66 | 2933.73 | *-C-H stretching* |
| 1654.92, 1631.78 | 1670.35, 1631.78 | -C=O stretching (Secondary amide) |
| 1087.85 | 1087.85 | -C-O stretching (5 member ring) |

**Table S13: Proton NMR peak assignment**

| Chemical Shift (δ ppm) | | Multiplicity | No of protons | Assignment |
| --- | --- | --- | --- | --- |
| Reference | Test |  |  |  |
| 11.93 | 11.96 | Singlet | 1 | 13 |
| 8.89-8.49 | 8.89-8.48 | Singlet | 2 | 28 |
| 7.90 | 7.89 | Triplet | 2 | 10 |
| 7.75-7.63 | 7.76--7.65 | Doublet | 2 | 5,8 |
| 4.21-4.17 | 4.21-4.17 | Multiplet | 1 | 15 |
| 3.86-3.82 | 3.86-3.83 | Doublet | 6 | 23, 25 |
| 3.76-3.65 | 3.76--3.65 | Multiplet | 3 | 12 |
| 3.34 | 3.33 | Singlet | 3 | 21 |
| 3.16-3.11 | 3.16-3.12 | Quartrate | 2 | 18 |
| 2.07-1.74 | 2.07-1.75 | Multiplet | 6 | 11,16 and 17 |

Table S14: ^13^C NMR Chemical shift Assignment for alfuzosin test and standard drug

| Chemical Shift (Delta ppm) | | No of carbon | Chemical Shift (Delta ppm) | | No of carbon |
| --- | --- | --- | --- | --- | --- |
| Reference drug | Test drug |  | Reference drug | Test drug |  |
| 172.5 | 172.46 | 14 | 77.71 | 77.69 | 15 |
| 161.04 | 161.03 | 2 | 68.52 | 68.49 | 18 |
| 155.08 | 155.07 | 4 | 56.26 | 56.25 | 23 |
| 151.77 | 151.75 | 26 | 56.03 | 56.01 | 25 |
| 146.58 | 146.56 | 6 | 47.23 | 47.21 | 12 |
| 136.27 | 136.26 | 7 | 35.94 | 35.92 | 10 |
| 105.05 | 105.07 | 8 | 35.68 | 35.66 | 21 |
| 101.51 | 101.5 | 27 | 29.93 | 29.88 | 16 |
| 99.25 | 99.26 | 5 | 26.96 | 26.93 | 11 |
|  | | 15 | 24.95 | 24.92 | 17 |

Table S15: Molecular ion peaks of Alfuzosin test and reference drugs

| Batch | Molecular Ion Peak (M + 1) |
| --- | --- |
| Reference drug | 390.00 |
| Test drug | 390.00 |

**Figure S1: Description of Large scale manufacturing process**

**A schematic diagram of manufacturing alfuzosin hydrochloride extended release tablets 10mg**

**Raw materials**

**In process**

**Analysis**

Alfuzosin Hydrochloride , Lactose Monohydrate, NF, Mono and Di Glycerides, NF, Magnesium Stearate, NF and Talc, NF

**Shifting**

**Dispensing**

Colloidal Silicon Dioxide, NF

Opacode black S-1-27794

Isopropyl Alcohol

**Dry mixing** (Jacketed rapid Mixer granulator)

Alfuzosin Hydrochloride , Lactose Monohydrate, NF, Mono and Di Glycerides, NF,

**Hot Melt granulation**

**Milling** (Oscillating Granulator equipped with

1. 2 I mm screen)

Colloidal silica (Aerosol 200)

**In-Process analysis**

Description

Identification

Assay

Blend unifonnity

**Blending** (Conta Blender)

Talc NF

**Lubrication** (Conta Blender)

Magnesium Stearate, NF

**In-Process Checks**

• Description

• Average weight Of tablets

• Individual weight variation

• Thickness

• Hardness • Friability

**Compression (Compression machine)**

**In-Process Checks**

Description

**Metal detection**

**(Metal Detector)**

**Tablet Imprinting**

**Acceptance Quality Level (AQL) test**

**Finished Product Analysis**

As per finished product specification

**Visual Inspection (Visual inspection Belt)**

**Packaging**
